# Patent Licensing and Capacity in a Cournot Model

**DOI:** 10.1007/s11151-022-09886-7

**Published:** 2022-11-17

**Authors:** Stefano Colombo, Luigi Filippini, Debapriya Sen

**Affiliations:** 1 Università Cattolica del Sacro Cuore, Largo Gemelli 1, 20123 Milan, Italy; 2 Ryerson University, 350 Victoria Street, Toronto, ON Canada

**Keywords:** Patent licensing, Cournot duopoly, Capacity constraint, D45

## Abstract

We consider the problem of patent licensing in a Cournot duopoly in which the innovator (patentee) is one of the firms and it is capacity constrained. We show that when the patentee can produce a relatively small (relatively large) quantity, it prefers licensing by means of a fixed fee (unit royalty). When the patentee can set two-part tariffs in the form of combinations of fixed fees and unit royalties, it charges a positive fixed fee if and only if it is limited to producing a relatively small quantity. We also show that with combinations of fixed fees and royalties, the royalty rate is lower than is true for the standard case.

## Introduction

We consider the problem of patent licensing in a Cournot duopoly in which the innovator (the patentee) is one of the competing firms and it is capacity constrained. When the capacity constraint is maximum (that is, the innovator cannot produce), the model coincides with the case of an outside patentee; when the capacity constraint is not binding, the model coincides with the case of an unconstrained inside patentee. Therefore, our model provides a bridge between the two cases usually considered in the literature: outside innovator and unconstrained inside innovator.

A capacity constraint is often relevant for the innovator. Pavitt et al. (1987), Acs and Audretsch (1990), OECD (2004), Marx et al. (2014), and Scholz (2017) provide evidence of the importance of small firms in generating technological innovations that are diffused by means of licenses. For example, Marx et al. (2014) explore the commercialization strategy in which a start-up temporarily enters the product market in order to establish the value of its innovation; ultimately, the entrant may switch to a strategy of cooperating with incumbents. Scholz (2017) emphasizes that—due to the increasing scarcity of raw materials that posit severe capacity constraints, especially for small firms—licensing agreements that delegate production (or part of production) to other firms are becoming widespread.^1^ In many cases the innovator is a small firm with limited production possibilities, which licenses its innovation to other firms.

The theoretical literature has rarely considered the role of a capacity constraint in determining the licensing choice of the patentee. Scholz (2017) analyses a vertical model where the upstream firms are capacity constrained, while the patentee is an outside innovator. Alderighi (2008) proposes a licensing method that involves a maximum authorized production for the licensee. However, to the best of our knowledge, the case of a capacity-constrained patentee has not been considered yet.

In a different context, Mukherjee (2001) examines the possibility of technology transfer when firms have pre-commitment strategies such as a capacity commitment or an incentive delegation under the assumption of fixed-fee licensing. Filippini (2005) suggests that restrictions on licensing contracts reduce the viability of technology transfers. Bagchi and Mukherjee (2011) show that an incumbent firm may hold excess capacity: not to deter entry but to extract a larger licensing fee.^2^

While the literature has shown that in the case of an outside (inside) innovator the fixed fee (unit royalty) is preferred by the patentee (Kamien & Tauman, 1986; Sen & Tauman, 2007; Wang, 1998), we show that a fixed fee is preferred by the patentee even if the patentee competes with the licensee—provided that the patentee is able to produce only a relatively small quantity. Therefore, a fixed fee might be preferred to a unit royalty even if the patentee is an insider. This happens both in the case of drastic and non-drastic innovation. We show that the results are driven by two parameters: *k*, the maximum quantity that can be produced by the capacity-constrained firm; and *c*, the cost reduction after the innovation (which is a measure of the innovation size).

Furthermore, we show that when the patentee can set two-part tariffs in the form of combinations of fixed fees and unit royalties, it charges a positive fixed fee if and only if the patentee can produce only a relatively small quantity due to the capacity constraint. We also show that with combinations of fixed fees and royalties, the royalty rate is lower than is true for the standard case.

The reason is the following: When the quantity that can be produced by the patentee is relatively small, it is better for the patentee to let the licensee expand its production, and then extract the extra profit of the licensee by means of a fixed fee. By contrast, when the patentee can produce a relatively large quantity, it benefits from expanding production and earns additional revenues from the royalties that it charges on the licensee’s output. Therefore, the patentee chooses a per-unit royalty.

The rest of the paper proceeds as follows: In Sect. 2 we introduce the model. In Sect. 3 we derive some preliminary results with regard to a constrained Cournot duopoly with asymmetric firms. In Sect. 4 we derive the equilibrium profits under licensing. In Sect. 5 we compare different licensing mechanisms. Section 6 discusses several extensions. Section 7 concludes.

## The Model

Consider a Cournot duopoly with two firms—1 and 2—with inverse demand: *p* = 1 − *q*_1_ − *q*_2_. Firm 1 (the patentee) has a cost-reducing innovation. The marginal cost of a firm is 0 with the innovation and *c* > 0 without the innovation where 0 < *c* < 1. Since firm 1 has the innovation, its marginal cost is 0. Firm 1 is constrained by capacity *k* ≥ 0, which is the maximum quantity that can be produced in a certain period of time, whereas firm 2 has no capacity constraint.

Note that when *k* = 0, the model coincides with the case of an outside innovator, whose production is zero (Kamien & Tauman, 1986). In this case, the non-producing inventor sets a fixed fee that absorbs all of the licensee’s profits: The inventor wants to maximize the licensee’s profits (and grab them via the fixed fee), and it does not want to distort production or sales (and the resulting profits) by means of a per-unit royalty (Kamien & Tauman, 1986).

On the other hand, when *k* is sufficiently high such that the capacity constraint is never binding in equilibrium (namely, *k* > 1), the model coincides with the case of an inside innovator with no capacity constraint (Wang, 1998). In this case, setting a per-unit royalty is preferred by the innovator, as it re-establishes cost asymmetry between the firms and, in addition, allows the patentee to get the license revenues (Wang, 1998).

Next, we introduce the distinction between drastic and non-drastic innovation (Arrow, 1962) Consider a monopolist that faces demand *p* = 1 − *Q*. The monopolist is not capacity constrained and has the cost-reducing innovation, so its marginal cost is 0. The monopoly price under marginal cost 0 is *p*_*M*_ ≡ 1/2. A cost-reducing innovation is *drastic* if the monopoly price *p*_*M*_ under the reduced cost (= 0) does not exceed *c* (the marginal cost without innovation); otherwise the innovation is *non-drastic*. Thus an innovation (which yields a marginal cost of 0) is drastic if *c* ≥ 1/2 and is non-drastic if *c* < 1/2.

### ***Remark***

If firm 1 is not capacity constrained and it has a drastic innovation, it has no incentive to license the innovation to firm 2 as without the innovation firm 2 drops out of the market and firm 1 obtains the monopoly profit under the reduced cost. However, this may not be the case when firm 1 is capacity constrained.

We consider three licensing policies:

(i) *Unit royalties* If firm 1 licenses the innovation to firm 2 with unit royalty *r* ≥ 0, firm 2 also has the cost-reducing innovation; and for every unit that firm 2 produces it has to pay *r* to firm 1. So the effective marginal cost of firm 2 is (0 + *r*) = *r*. Firm 2’s marginal cost without the innovation is *c*, so the unit royalties that are acceptable to firm 2 must satisfy *r* ≤ *c*.
(ii) *Fixed fees* If firm 1 licenses the innovation to firm 2 with a fixed fee *f* ≥ 0, firm 2 has the cost-reducing innovation, and it pays the fee *f* upfront to firm 1.
(iii) *Combinations of unit royalties licensing and fixed fees* If firm 1 licenses the innovation to firm 2 with the use of a policy (*r*, *f*) that has a unit royalty *r* ≥ 0 and a fixed fee *f* ≥ 0, firm 2 has the cost-reducing innovation; it pays the fee *f* upfront to firm 1; and for every unit that it produces, it has to pay *r* to firm 1. So the effective marginal cost of firm 2 is (0 + *r*) = *r*.

Since firm 1 has the cost-reducing innovation, its marginal cost is 0. If firm 2 does not have the innovation, its marginal cost is *c*. Therefore, *c* can be interpreted as the innovation size. If firm 2 has the innovation under a fixed fee policy, its marginal cost is 0. If firm 2 has the innovation under a policy that has royalty *r* (either a royalty policy or a policy that is a combination of royalty and fee), then the effective marginal cost of firm 2 is *r*.

The strategic interaction between firms 1 and 2 is modeled as the three-stage licensing game *G*: In stage 1 of *G*, firm 1 decides whether to licenses its innovation to firm 2 or not and offers a licensing policy to firm 2; in stage 2 firm 2 decides whether to accept the policy or not; in stage 3, firms 1 and 2 compete in the Cournot duopoly, and payments are made according to the policy.

## Cournot Duopoly ***D***^***k***^(***r***)

For 0 ≤ *r* ≤ *c* and *k* ≥ 0, denote by *D*^*k*^(*r*) the Cournot duopoly in which firm 1 has marginal cost 0 and capacity constraint *k*; firm 2 has marginal cost *r* and no capacity constraint. In particular note that with respect to the marginal cost of firm 2, *r* = *c* corresponds to the situation where firm 2 does not have the innovation.

Thus, if firm 2 has the innovation under a fixed fee policy, the resulting Cournot duopoly is *D*^*k*^(0). If firm 2 has the innovation under a policy that has royalty *r*, it is *D*^*k*^(*r*). If firm 2 does not have the innovation, the resulting Cournot duopoly is *D*^*k*^(*c*).

To determine the optimal licensing policies for firm 1, it is therefore useful to determine the equilibrium outcomes of *D*^*k*^(*r*) for all 0 ≤ *r* ≤ *c* and *k* ≥ 0. When there is no capacity constraint, the quantities that are produced by firms 1,2 in the unique (Cournot-Nash) equilibrium are $$\overline{q}_{1} (r) = \left\{ {\begin{array}{*{20}l} {{{(1 + r)} \mathord{\left/ {\vphantom {{(1 + r)} 3}} \right. \kern-\nulldelimiterspace} 3}} \hfill \\ {{1 \mathord{\left/ {\vphantom {1 2}} \right. \kern-\nulldelimiterspace} 2}} \hfill \\ \end{array} } \right.$$ $$\begin{array}{*{20}l} {if} \hfill & {0 \le r < {1 \mathord{\left/ {\vphantom {1 2}} \right. \kern-\nulldelimiterspace} 2}} \hfill \\ {if} \hfill & {r \ge {1 \mathord{\left/ {\vphantom {1 2}} \right. \kern-\nulldelimiterspace} 2}} \hfill \\ \end{array}$$ and $$\overline{q}_{2} (r) = \left\{ {\begin{array}{*{20}l} {{{(1 - 2r)} \mathord{\left/ {\vphantom {{(1 - 2r)} 3}} \right. \kern-\nulldelimiterspace} 3}} \hfill \\ 0 \hfill \\ \end{array} } \right.$$ $$\begin{gathered} \begin{array}{*{20}c} {if} & {0 \le r < {1 \mathord{\left/ {\vphantom {1 2}} \right. \kern-\nulldelimiterspace} 2}} \\ \end{array} \hfill \\ \begin{array}{*{20}c} {if} & {r \ge {1 \mathord{\left/ {\vphantom {1 2}} \right. \kern-\nulldelimiterspace} 2}} \\ \end{array} \hfill \\ \end{gathered}$$; the equilibrium price is $$\overline{p}(r) = \left\{ \begin{gathered} {{(1 + r)} \mathord{\left/ {\vphantom {{(1 + r)} 3}} \right. \kern-\nulldelimiterspace} 3} \hfill \\ {1 \mathord{\left/ {\vphantom {1 2}} \right. \kern-\nulldelimiterspace} 2} \hfill \\ \end{gathered} \right.$$ $$\begin{gathered} \begin{array}{*{20}c} {if} & {0 \le r < {1 \mathord{\left/ {\vphantom {1 2}} \right. \kern-\nulldelimiterspace} 2}} \\ \end{array} \hfill \\ \begin{array}{*{20}c} {if} & {r \ge {1 \mathord{\left/ {\vphantom {1 2}} \right. \kern-\nulldelimiterspace} 2}} \\ \end{array} \hfill \\ \end{gathered}$$; and the equilibrium profits are $$\overline{\pi }_{1} (r) = \left\{ \begin{gathered} {{(1 + r)^{2} } \mathord{\left/ {\vphantom {{(1 + r)^{2} } 9}} \right. \kern-\nulldelimiterspace} 9} \hfill \\ {1 \mathord{\left/ {\vphantom {1 4}} \right. \kern-\nulldelimiterspace} 4} \hfill \\ \end{gathered} \right.$$ $$\begin{array}{*{20}l} {if} \hfill & {0 \le r < {1 \mathord{\left/ {\vphantom {1 2}} \right. \kern-\nulldelimiterspace} 2}} \hfill \\ {if} \hfill & {r \ge {1 \mathord{\left/ {\vphantom {1 2}} \right. \kern-\nulldelimiterspace} 2}} \hfill \\ \end{array}$$ and $$\overline{\pi }_{2} (r) = \left\{ {\begin{array}{*{20}l} {{{(1 - 2r)^{2} } \mathord{\left/ {\vphantom {{(1 - 2r)^{2} } 9}} \right. \kern-\nulldelimiterspace} 9}} \hfill \\ 0 \hfill \\ \end{array} } \right.$$ $$\begin{array}{*{20}l} {if} \hfill & {0 \le r < {1 \mathord{\left/ {\vphantom {1 2}} \right. \kern-\nulldelimiterspace} 2}} \hfill \\ {if} \hfill & {r \ge {1 \mathord{\left/ {\vphantom {1 2}} \right. \kern-\nulldelimiterspace} 2}} \hfill \\ \end{array}$$.

### Lemma 1

*For any* 0 < *c* < 1, *the Cournot duopoly D*^*k*^(*r*) *has a unique* (*Cournot-Nash*) *equilibrium*. *If the capacity k exceeds* $\overline{q}_{1} (r)$, *the equilibrium outcome is the same as the case with no capacity constraint*. *Otherwise, the capacity constraint is binding, and firm* 1 *exhausts its capacity* (*that is, q*_1_ = *k*).

### ***Proof***

See the online Appendix.^3^ □

For the Cournot duopoly *D*^*k*^(*r*), denote the equilibrium price by *p*^*k*^(*r*); quantities of firms 1,2 by *q*_1_^*k*^(*r*), *q*_2_^*k*^(*r*); and profits by φ_1_^*k*^(*r*), φ_2_^*k*^(*r*).

## Equilibrium Profits

*No license*. When firm 1 does not license the innovation, the resulting Cournot duopoly is *D*^*k*^(*c*), where firm 1 obtains Cournot profit φ_1_^*k*^(*c*).

*Unit royalty policy.* When firm 1 licenses the innovation to firm 2 with a unit royalty *r* ≥ 0, the Cournot duopoly game *D*^*k*^(*r*) is played where the Cournot quantity of firm 2 is *q*_2_^*k*^(*r*). So for firm 1, the licensing revenue from royalty is *rq*_2_^*k*^(*r*). The payoff of firm 1 is the sum of its Cournot profit and licensing revenue, which is given by:

1 $$\uppi ^{k}_{R} \left( r \right) \, = \, {\upvarphi }_{1}^{k} \left( r \right) \, + rq_{2}^{k} \left( r \right) \, . $$

Recall that no royalty with *r* > *c* is acceptable to firm 2. So under the unit royalty policy, the problem of firm 1 is to choose *r* (0 ≤ *r* ≤ *c*) so as to maximize π^*k*^_*R*_(*r*) given in (1). We also need to compare the payoff from optimal royalty policy with φ_1_^*k*^(*c*) to see whether licensing by means of a royalty is superior than not licensing.

*Fixed-fee policy.* When firm 1 licenses the innovation to firm 2 with fixed fee *f* ≥ 0, the resulting Cournot duopoly is *D*^*k*^(0) in which firm 2 obtains the Cournot profit φ_2_^*k*^(0). If firm 2 refuses to have a license, the resulting Cournot duopoly is *D*^*k*^(*c*) in which firm 2 obtains the Cournot profit φ_2_^*k*^(*c*). Therefore the maximum fixed fee that firm 1 can set is φ_2_^*k*^(0) − φ_2_^*k*^(*c*) (provided that this is non-negative), which makes firm 2 just indifferent between accepting and rejecting. So the payoff that firm 1 has under the fixed-fee policy has two parts: (i) firm 1's Cournot profit φ_1_^*k*^(0); and (ii) fixed fee φ_2_^*k*^(0) − φ_2_^*k*^(*c*). This payoff is:

2 $$\uppi ^{k}_{F} = \, {\upvarphi }_{1}^{k} \left( 0 \right) \, + \, {\upvarphi }_{2}^{k} \left( 0 \right) \, - {\upvarphi }_{2}^{k} \left( c \right) \, . $$

We need to compare this payoff with φ_1_^*k*^(*c*) to see whether licensing by means of a fixed fee is superior than not licensing.

*Combinations of unit royalties and fixed fees policy.* Suppose that firm 1 licenses the innovation to firm 2 with the use of a licensing policy (*r*, *f*) where *r* (0 ≤ *r* ≤ *c*) is the unit royalty and *f* ≥ 0 is the fixed fee that firm 2 has to pay to firm 1. If firm 2 accepts this policy, it obtains the Cournot profit φ_2_^*k*^(*r*). If it rejects, it operates with marginal cost *c* and obtains the Cournot profit φ_2_^*k*^(*c*). So for any *r*, the maximum fixed fee that firm 1 can set is:

3 $$ f = {\upvarphi }_{2}^{k} \left( r \right) \, - \, {\upvarphi }_{2}^{k} \left( c \right) \, . $$

Under the licensing policy (*r*, *f*), the payoff of firm 1 has three parts: (i) its Cournot profit φ_1_^*k*^(*r*); (ii) its royalty revenue *rq*_2_^*k*^(*r*); and (iii) the fixed fee *f* that is given by (3). When *f* is chosen optimally as in (1), the payoff of firm 1 as function of *r* is:

4 $$\uppi ^{k}_{RF} \left( r \right) \, = \, {\upvarphi }_{1}^{k} \left( r \right) \, + rq_{2}^{k} \left( r \right) \, + \, {\upvarphi }_{2}^{k} \left( r \right) \, - \, {\upvarphi }_{2}^{k} \left( c \right) . $$

As the fixed fee *f* is chosen optimally for any *r*, under combinations of unit royalties and fees the problem of firm 1 is to choose *r* (0 ≤ *r* ≤ *c*) so as to maximize π^*k*^_*RF*_(*r*) that is given in (4). We also need to compare the payoff from the optimal combination with φ_1_^*k*^(*c*) to see whether such a policy is superior than not licensing.

For the analysis it will be convenient first to characterize the optimal combinations of unit royalties and fees:

*Optimal combinations of unit royalties and fixed fees* When the unit royalty is *r*, the resulting Cournot duopoly is *D*^*k*^(*r*) in which firm 1 has marginal cost 0 and firm 2 has (effective) marginal cost *r*. Therefore Cournot profits are: φ_1_^*k*^(*r*) = *p*^*k*^(*r*)*q*_1_^*k*^(*r*) and φ_2_^*k*^(*r*) = [*p*^*k*^(*r*) − *r*]*q*_2_^*k*^(*r*).

Using this in (4) and denoting *Q*^*k*^(*r*) = *q*_1_^*k*^(*r*) + *q*_2_^*k*^(*r*) (the total quantity), we have:

5 $$ \begin{aligned}\uppi ^{k}_{RF} \left( r \right) & = p^{k} \left( r \right)q_{1}^{k} \left( r \right) \, + rq_{2}^{k} \left( r \right) \, + \, \left[ {p^{k} \left( r \right) \, - r} \right]q_{2}^{k} \left( r \right) \, - \, {\upvarphi }_{2}^{k} \left( c \right) \, = p^{k} \left( r \right)\left[ {q_{1}^{k} \left( r \right) \, + q_{2}^{k} \left( r \right)} \right] \, - \, {\upvarphi }_{2}^{k} \left( c \right) \\ & = p^{k} \left( r \right)Q^{k} \left( r \right) \, - \, {\upvarphi }_{2}^{k} \left( c \right) \, = p^{k} \left( r \right)\left[ {1 \, - p^{k} \left( r \right)} \right] \, - \, {\upvarphi }_{2}^{k} \left( c \right) \, . \\ \end{aligned} $$

For any price *p* (0 ≤ *p* ≤ 1), let φ_*M*_(*p*) = *p*(1 − *p*) be the profit of the monopolist at price *p* which has marginal cost 0. Observe from (5) that:

6 $$\uppi ^{k}_{RF} \left( r \right) \, = {\upvarphi }_{M} \left( {p^{k} \left( r \right)} \right) - {\upvarphi }_{2}^{k} \left( c \right) \, . $$

Since φ_2_^*k*^(*c*) is a constant that is not affected by *r*, by (4) the problem of firm 1 is to choose *r* so as to maximize φ_*M*_(*p*^*k*^(*r*)). Note that φ_*M*_(*p*) is increasing for *p* < 1/2 and is decreasing for *p* > 1/2; and its unique maximum is attained when *p* equals *p*_*M*_ ≡ 1/2: the monopoly price with marginal cost 0. Let φ_M_^*^ ≡ φ_*M*_(*p*_*M*_) = 1/4 (the monopoly profit at marginal cost 0). From (4), the maximum possible payoff that firm 1 can obtain is φ_M_^*^ − φ_2_^*k*^(*c*): the monopoly profit with marginal cost 0 by leaving firm 2 its reservation profit φ_2_^*k*^(*c*).

Proposition 1 characterizes the optimal combinations of unit royalties and fixed fees:

### **Proposition 1**

*When firm* 1 *uses combinations of unit royalties and fixed fees, the optimal licensing policies are as follows:*

1. *Suppose that the innovation is non-drastic: c* < 1/2. *If k* < *c,*^4^* the unique optimal policy for firm* 1 *is to license the innovation to firm* 2 *with the use of a unit royalty r* = *k and a positive fixed fee. If k* ≥ *c, the unique optimal policy is to license the innovation to firm* 2 *with the use of a pure royalty policy* (*zero fixed fee*) *with a unit royalty r* = *c*.
2. *Suppose that the innovation is drastic: c* ≥ 1/2. *If k* < *Q*_*M*_ ≡ 1/2 (*the monopoly output with marginal cost* 0)*, the unique optimal policy for firm* 1 *is to license the innovation to firm* 2 *with the use of a unit royalty r* = *k and a positive fixed fee. If k* ≥ 1/2*, it is optimal for firm* 1 *to not license the innovation and instead to use it exclusively to obtain the monopoly profit*.

### ***Proof***

See the Appendix. □

**Fig. 1:**
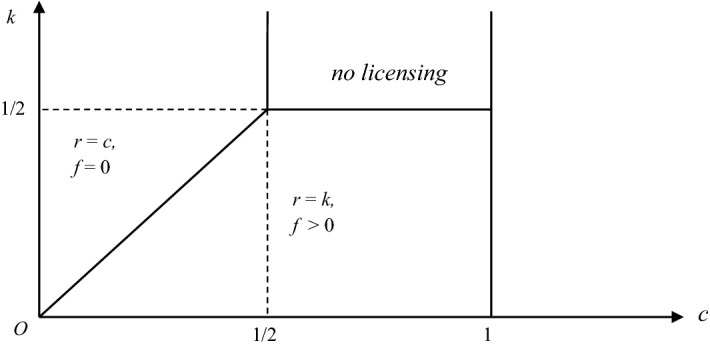
Optimal combinations of unit royalties and fixed fees

The optimal licensing policy for the innovator—and the equilibrium price, quantity, and profits—depends on the model parameters’ values: the innovation size/original unit cost, *c*; and the maximum quantity of output that the innovator can produce, *k* (see Fig. 1). In particular, a positive fixed fee emerges only in the parameter area where *k* is low enough—where the patentee can produce only a relatively small quantity—for both the case of drastic innovation and of non-drastic innovation. Moreover, we show that with combinations of fixed fees and royalties, the royalty rate is lower than the standard case.

For example, consider the case of non-drastic innovation: It is well-know that, without a capacity constraint, the optimal royalty would be *r* = *c*. However, with a capacity constraint, we observe *r* = *k* < *c*. Therefore, the capacity constraint modifies the optimal combinations of royalty and fee.

In particular, differently from the case of optimal two-part tariffs with no-capacity constraint (see for example Faulì-Oller & Sandonìs, 2002, and Colombo, 2012), there are situations where the optimal royalty rate is smaller than the marginal cost reduction that is due to the process innovation. Furthermore, in these situations, the fixed fee is positive, rather than zero. This happens when *k* is small enough: The patentee is able to produce only a relatively small quantity.

The explanation is the following: When the patentee’s output is limited and relatively small (*k* is low), it is better for the patentee to allow the licensee to expand production (by means of a lower royalty, which is equal to *k*^5^), and then extract the licensee’s extra profit by means of a positive fixed fee. Note that this explanation is similar to the argument in Kamien and Tauman (1986). Indeed, when *k* is relatively small, our model is similar to a model with an outside patentee.

In contrast, when the patentee is not strongly constrained by the capacity constraint (*k* is high), it is better for the patentee to expand its own production and also receive additional revenues from the royalty on the licensee’s output.^6^ Therefore, the per-unit royalty is maximized—*r* = *c*—and the fixed fee is zero.^7^ The intuition in this case is similar to Wang (1998). Indeed, when *k* is high, our model is similar to a model with an inside patentee with no capacity constraint.

### ***Remark***

Note that the set of licensing policies with combinations of fixed fees and royalties include as special cases the policies that have only fixed fees or only royalties. Therefore:

(i) For cases where not licensing is superior to combinations of unit royalties and fixed fees for firm 1, not licensing must be also superior to only fixed fees or only unit royalties.
(ii) For cases where the optimal combination has only a royalty and no fixed fee and such a policy is also superior to not licensing, this policy must also be the optimal unit royalty policy as well, and it must be also superior to pure fixed fees.

The following corollary is immediate from Proposition 1:

### **Corollary 1**

1. *If the innovation is non-drastic* (*c* < 1/2) *and k* ≥ *c, the unique optimal unit royalty policy for firm* 1 *has r* = *c*. *This policy is superior to not licensing and to any pure fixed-fee policy.*
2. *If the innovation is drastic* (*c* ≥ 1/2) *and k* ≥ 1/2*, not licensing is superior to both unit royalty policies and to fixed-fee policies; It is optimal for firm* 1 *to not license the innovation and to use it exclusively to obtain the monopoly profit*.

*Optimal unit royalty policies* In view of Corollary 1, to completely characterize optimal pure royalty policies, we need to find optimal pure royalty policies for the parameter space where *k* < 1/2 and *k* < *c*. The next proposition presents the result:

### **Proposition 2**

*The optimal unit royalty policies for firm* 1 *are as follows:*

1. *Suppose that the innovation is non-drastic: c* < 1/2. *Then the unique optimal policy for firm* 1 *is to license the innovation to firm* 2 *with the use of a unit royalty r* = *c*.
2. *Suppose that the innovation is drastic: c* ≥ 1/2. *If k* < 1/2*, the unique optimal policy for firm* 1 *is to license the innovation to firm* 2 *with the use of a unit royalty r* = 1/2. *If k* ≥ 1/2*, it is optimal for firm* 1 *to not license the innovation and to use it exclusively to obtain the monopoly profit*.

### *Proof*

See the Appendix. □

**Fig. 2:**
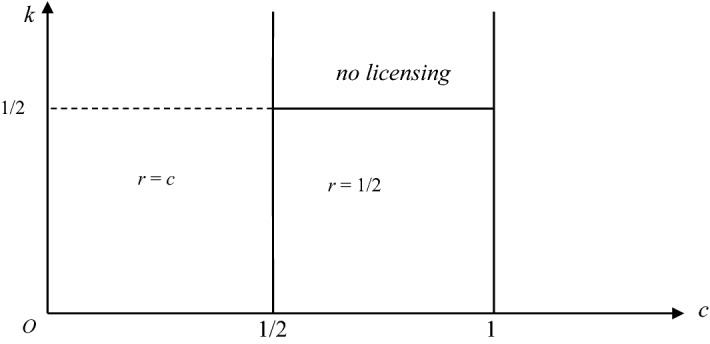
Optimal pure royalty policies

Figure 2 presents optimal pure royalty policies for firm 1 in the (*c*, *k*) plane. Note that this result coincides with Wang (1998) when the innovation is non-drastic: When the fixed fee is zero, it is optimal for the patentee to set the highest per-unit royalty—*r* = *c*—and to license to the rival. Therefore, the capacity constraint plays no role when the innovation is non-drastic.

By contrast, when the innovation is drastic and *k* is smaller than 1/2, the patentee cannot produce the monopoly output due to the capacity constraint. In this case, it is better for the patentee to produce as much as possible and to get extra revenues from the per-unit royalty that is charged to the licensee. Note that this result is different from Wang (1998), and highlights the role that is played by the capacity constraint. Finally, when *k* is greater than 1/2, the patentee produces the monopoly outcome, and it does not license, as in Wang (1998).

*Optimal fixed-fee policies* By Corollary 1, if *c* ≥ 1/2 (drastic innovations) and *k* ≥ 1/2, not licensing is superior to combinations of unit royalties and fixed fees, so not licensing is also superior to fixed-fee policies. To characterize optimal fixed-fee policies completely, we examine the rest of the regions:

### **Proposition 3**

*The unique optimal fixed-fee policy for firm* 1 *is to set f* = φ_2_^*k*^(*r*) − φ_2_^*k*^(*c*), *and it has the following properties.*

1. *If c* < 2/5, *the fixed-fee policy is superior to not licensing for all k.*
2. *If* 2/5 < *c* < 2/3, *there is a decreasing function k*_0_(*c*) *such that the fixed-fee policy is superior to not licensing if k* < *k*_0_(*c*) *and not licensing is superior to the fixed fee policy if k* > *k*_0_(*c*).
3. *If* 2/3 < *c* < 1, *the fixed-fee policy is superior to not licensing if k* < 1/3, *and not licensing is superior to the fixed-fee policy if k* > 1/3.

### ***Proof***

See the Appendix. □

**Fig. 3:**
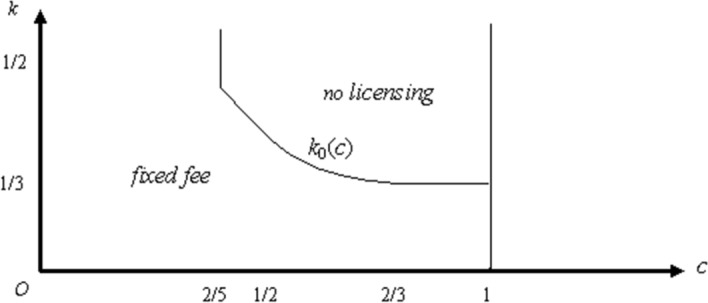
Optimal pure fixed fee policies

Figure 3 presents optimal fixed fee royalty policies for firm 1 in the (*c*, *k*) plane. If *c* < 2/5, the fixed fee is superior to not licensing for any *k*. If *c* ≥ 2/5, the fixed fee is superior to not licensing if *k* is below *k*_0_(*c*), and not licensing is superior if *k* is above *k*_0_(*c*).

It is interesting to observe that when *c* < 2/5, we are back to the result in Wang (1998, Proposition 1): Licensing by means of a fixed fee is always better than no licensing. However, for greater values of *c*, the capacity constraint starts to play a role in determining the optimal choice of the patentee between fixed fee and no licensing. Indeed, when *k* is low, the patentee’s output is relatively small. Therefore, firm 1 cannot benefit so much from the marginal cost differential: It is better for firm 1 to allow firm 2 to expand its production (by licensing the cost-reducing innovation) and extract the extra profits of firm 2 by means of the fixed fee. By contrast, when *k* is high, firm 1’s output is large. Therefore, firm 1 keeps the marginal costs difference by not licensing the innovation to the rival.

## Comparing Unit Royalty and Fixed Fee Policies

We can now compare the optimal royalty and fixed-fee policies.

### **Proposition 4**

*For firm* 1*, fixed fee, unit royalty, and not licensing compare as follows:*

1. *Suppose that* 0 < *c* < 1/2. *A unit royalty policy is superior to both a fixed fee and not licensing if k* > *c*/2; *and fixed fee is superior to both a unit royalty and not licensing if k* < *c*/2.
2. *Suppose that* 1/2 ≤ *c* < 1*. There is a function k*_1_(*c*) *such that a fixed fee is superior to both a unit royalty and not licensing if k* < *k*_1_(*c*); a *unit royalty policy is superior to both a fixed fee and not licensing if k*_1_(*c*) < *k* < ½; *and not licensing is superior to both a unit royalty policy and a fixed fee if k* > 1/2.

### ***Proof***

See the Appendix. □

**Fig. 4:**
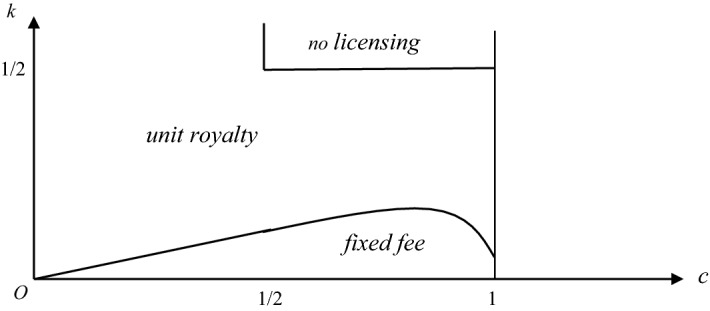
Comparing unit royalty, fixed fee, and not licensing policies

Figure 4 summarizes the above discussion, by indicating the parameter spaces where each licensing mechanism emerges in equilibrium. Figure 4 shows that a fixed fee is superior to unit royalties (and to no licensing), provided that the patentee is able to produce only a relatively small quantity—both for the case of drastic innovation and for non-drastic innovation.

As was argued above, when the quantity that can be produced by the patentee is relatively small, it is better for the patentee to let the licensee expand the latter’s production and then extract the extra profit of the licensee by means of a fixed fee. This result is similar to Kamien and Tauman (1986). Indeed, when *k* is relatively low, our model is similar to a licensing game with an outside innovator.

By contrast, when the patentee can produce a relatively large amount of output, it gets additional revenues from the royalties that are charged on the licensees’ output. In this case, a per-unit royalty is better than a fixed fee from the patentee’s perspective. This result resembles that in Wang (1998). Indeed, when *k* is relatively large, our model is similar to a licensing game with an inside unconstrained innovator.

## Extensions

In this section, we briefly discuss some extensions of the basic model. The mathematical derivations are relegated in the Technical Appendix.

### Stackelberg Competition

In this subsection, we consider the case where the firms compete *a lá* Stackelberg. In particular, firm 1 moves first in the quantity-setting stage, whereas firm 2 moves second. We consider only non-drastic innovation, which in the case of Stackelberg competition amounts to assuming that *c* < 1/3 (Filippini, 2005).

We state the following proposition, which extends Proposition 1 to the case of Stackelberg competition:

#### **Proposition 5**

*When firm* 1 *is the leader in a Stackelberg game and uses combinations of unit royalties and fixed fees, the optimal licensing policies are as follows: If k* < *c, the unique optimal policy for firm* 1 *is to license the innovation to firm* 2 *with the use of the unit royalty r* = *k and a positive fixed fee. If k* ≥ *c, the unique optimal policy is to license the innovation to firm* 2 *with the use of a pure royalty policy* (*zero fixed fee*) *with the unit royalty r* = *c*.

### Incomplete Cost Reduction

In this subsection, we consider the case where the process innovation does not drive the marginal costs to zero. We consider two situations: *i*) the firms are symmetric: Under innovation, the marginal costs of both firms are *c* – *ε*, whereas without innovation they are equal to *c*; and *ii*) the firms are asymmetric: Under innovation the marginal costs of firm 2 are *c* – *ε* and those of firm 1 are 0–1 is more efficient than is firm 2 in using the innovation—whereas without innovation the marginal costs are equal to *c*. We consider only non-drastic innovation, which in this case amounts requiring that *c* < 1 – *ε*, with *c* > *ε* > 0.

For both case i and case *ii*, we can state the following proposition, which is similar to Proposition 4:

#### **Proposition 6**

*For firm* 1*, fixed fee, unit royalty and not licensing compare as follows: Suppose that* 0 < *c* < 1 – *ε*: *A unit royalty policy is superior to both a fixed fee and not licensing if k* > *ε*/2, *and a fixed fee is superior to both a unit royalty and not licensing if k* < *ε*/2.

### Firm 2 with Bargaining Power

In this subsection, we consider the case where the licensee has bargaining power. In the basic model, we have assumed that all of the bargaining power belongs to the patentee (firm 1), following the literature. However, some research has investigated the possibility of bargaining between the patentee and the licensee about the licensing contracts; see, for example, Watanabe and Muto (2008); and Kishimoto and Muto (2012).

While a complete analysis of bargaining seems intractable, even numerically, in the context of a capacity-constrained patentee, we analyse the opposite extreme of the spectrum: a situation where all of the bargaining power belongs to the licensee (firm 2). We consider only the case of a non-drastic innovation. We can state the following proposition:

#### **Proposition 7**

*For firm *2*, a fixed fee, a unit royalty, and not licensing compare as follows. Suppose that* 0 < *c* < 1/2. *A unit royalty policy is at least as good as a fixed fee and is superior to not licensing*.

Therefore, when the bargaining power belongs to firm 2, unit-royalty is never inferior to fixed fee for the licensee. Indeed, because of the bargaining power and whatever the level of *k* is, firm 2 can reduce the equilibrium royalty *r* to be paid to firm 1 more than the fixed fee *F*, thus making per-unit royalty licensing more profitable (for firm 2) than fixed fee licensing. Note that this result and its intuition are similar to Proposition 3 in Kishimoto and Muto (2012).

### Excess Capacity Licensing

In this subsection, we suppose that firm 2 has excess capacity that it can license to firm 1.^8^ The game has a Stackelberg structure: In stage 0 of *G*, firm 2 decides whether to license its excess capacity to firm 1 or not and offers a licensing policy to firm 1 (only fixed-fee licensing is considered at this stage^9^); in stage 1 of *G*, firm 1 decides whether to license its innovation to firm 2 or not and offers a licensing policy to firm 2; in stage 2 firm 2 decides whether to accept the policy or not; in stage 3, firms 1 and 2 compete in the Cournot duopoly, and payments are made according to the policy.

Note that if in stage 0 firm 2 does not license its excess capacity, we are back to the model that we have already solved in this paper; whereas if firm 2 licenses its excess capacity, the model coincides with Wang (1998). We state the following proposition:

#### **Proposition 8**

*Firm 2 licenses its excess capacity: when* 0 < *c* < 1/2 *(a non-drastic innovation) and* (– 1 + 5*c*)/3 < *k* < (1 – 2*c*)/3*; or when* 1/2 < *c* < 1 *(a drastic innovation) and k* < 1/2; *otherwise firm 2 does not license its excess capacity.*

**Fig. 5:**
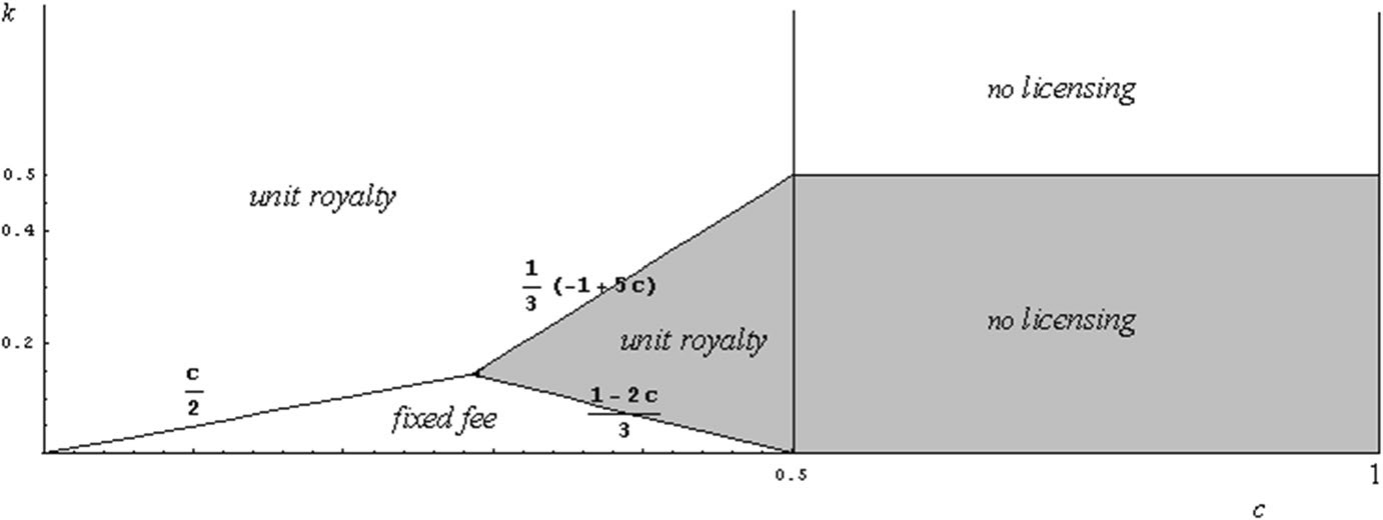
Excess capacity licensing

Figure 5 illustrates Proposition 8. The grey area indicates the parameter set where excess capacity licensing by firm 2 occurs. In Fig. 5 we also indicate the licensing mechanism (if any) that is adopted by firm 1 to license the process innovation. The process innovation licensing equilibrium is obtained by applying directly Proposition 4 when there is not excess capacity licensing by firm 2, and the Wang (1998) results when there is excess capacity licensing.

It can be observed that capacity licensing is beneficial for firm 2 when *c* is high enough (and, clearly, when *k* is not too high, because when *k* is relatively high the capacity constraint of firm 1 is not binding in equilibrium). This happens because when *c* is high, the advantage of firm 1 over firm 2 is large.^10^ This is true especially when firm 1 is not capacity constrained. Therefore, firm 2 might extract a larger fixed fee from firm 1 in exchange for capacity.

### Bertrand Competition

In this subsection, we consider price competition rather than quantity competition. In particular, suppose that the demand function is *Q* = 1 − *p*, where *p* is the lowest price: Since the goods are homogenous, only the firm that sets the lowest price serves the consumers.^11^ We consider only non-drastic competition (*c* < 1/2).^12^

Consider first no licensing: Since the marginal cost of firm 2 is *c* and that of firm 1 is 0, the equilibrium price is *c*. If the capacity constraint is not binding, firm 1 sells an amount 1 – *c* and earns profits that are equal to *c*(1 – *c*), whereas firm 2 does not sell and gets zero. Suppose now that the capacity constraint is binding: *k* < 1 – *c*. The equilibrium price is still *c*; but firm 1 produces *k* and receives profits that are equal to *ck*, whereas firm 2 produces 1 – *c* – *k* and gets profits *c*(1 – *c* – *k*).

Now we introduce licensing: First, we show that when there is a positive royalty, the fixed fee must be zero. Indeed, the royalty *r* is the marginal cost for firm 2. Under Bertrand competition, the equilibrium price is *r*, and hence the profits of firm 2 are zero. It follows that no positive fixed fee could be accepted by firm 2. In other words, a two-part tariff never arises under Bertrand competition with homogenous goods.

Consider a pure royalty: When the capacity constraint is not binding – *k* ≥ 1 – *c* – the profits of firm 1 are *r*(1 – *r*); and firm 2 sells nothing. Since the profits of firm 1 are increasing in *r* (recall that *r* < *c* < 1/2), firm 1 sets *r* = *c*, and receives *c*(1 – *c*) as in the case of no license. Therefore, without the capacity constraint, firm 1 prefers not to license. Now consider the capacity constraint: *k* < 1 – *c*. If firm 1 licenses by using a royalty *r*, its profits are *rk* + *r*(1 – *r* – *k*) = *r*(1 – *r*). Hence, firm 1 sets *r* = *c*, and receives *c*(1 – *c*), which is greater than *ck*.

Finally, consider a fixed fee. Since the firms have the same marginal cost (which is zero), the equilibrium price is zero. Therefore, the maximum fixed fee that could be required from firm 2 is zero as well. It follows that the profits of firm 1 are zero.

The following proposition summarizes the above discussion:

#### **Proposition 9**

*When firm* 1 *and firm* 2 *compete in prices, the optimal licensing policies are as follows: If k* < (1 *– c*)*, the unique optimal policy for firm* 1 *is to license the innovation to firm* 2 *with the use of a pure royalty policy* (a *zero fixed fee*) *with the unit royalty r* = *c. If k* ≥ (1 *– c*)*, it is optimal for firm* 1 *to not license the innovation*.

Proposition 9 highlights the difference between price and quantity competition in our context: With Cournot competition, even when firm 2 has higher costs than firm 1, firm 2 continues to sell as long as its cost is below the monopoly price. As firm 1 cannot prevent this outcome, it finds it profitable to license via a unit royalty both when there is a capacity constraint and when there is not (see Wang, 1998). By contrast, in the case of Bertrand competition, firm 2 is prevented (by firm 1’s limit pricing) from selling as long as it is less efficient than firm 1: Without a capacity constraint, firm 1 charges a limit price of *p* = *c*, and it chooses not to license the innovation. Licensing becomes profitable for firm 1 if and only if the capacity constraint holds; and in this case the optimal mechanism is a pure royalty with *r* = *c*.

## Conclusions

We introduce a capacity constraint for an innovator and we discuss optimal licensing in a Cournot duopoly. Our model links the two models that are usually considered in the literature: an outside innovator, and an unconstrained inside innovator. We consider unit royalties, fixed fees, and combinations of unit royalties and fixed fees.^13^ We show that a fixed fee is used if and only if the patentee is able to produce only a relatively small quantity (because of a binding capacity constraint). Therefore, a fixed fee might be preferred by the patentee even if the innovator competes with the licensee. When the patentee sets a two-part tariff by combining fixed fees and unit royalties, it charges a positive fixed fee if and only if the patentee is able to produce only a relatively small quantity. Furthermore, the optimal royalty rate in the optimal two-part tariff is lower than the standard case.

The results are driven by two key parameters: *i*) the maximum quantity that can be produced by the capacity-constrained firm; and *ii*) the innovation size.^14^

## Supplementary information

The Appendix is available at: https://sites.google.com/view/stefanocolombo.

## Footnotes

1 OECD (2004) stresses that “[innovating] *firms lack the complementary assets, such as marketing and manufacturing, which are necessary to successfully commercialise their inventions*” (p.16). Pavitt et al. (1987), by analyzing the size distribution of innovating firms in UK after the Second World War, show that smaller firms are more likely to commercialize innovations than are bigger firms. More recently, McClellan et al. (2020) suggest that—when developing monoclonal antibodies as a treatment for COVID-19—total capacity is split between companies that employ an in-house manufacturing network and others that act solely as contract manufacturers.

2 As capacity constraint is a specific form of decreasing returns to scale, our work also relates to the literature on patent licensing under returns to scale (Sen & Stamatopoulos, 2009, 2016, 2019).

3 The Appendix is available at: https://sites.google.com/view/stefanocolombo.

4 Note that—because of the specific parameterization of the units of the demand function—this comparison is mathematically valid, even though *k* is a capacity measure that is expressed in physical units and *c* is a cost measure that is expressed in monetary units per physical unit.

5 Indeed, firm 1 chooses *r* in order to maximize φ_*M*_ (*p*^*k*^(*r*)). When the capacity constraint is not binding, φ_*M*_ (*p*^*k*^(*r*)) strictly increases with *r*, and therefore *r* = *c*, and the fixed fee is zero. However, when the capacity constraint is binding – *q*_1_ = *k* – φ_*M*_ (*p*^*k*^(*r*)) increases with *r* when *r* < *k* and decreases when *r* > *k*. Therefore, firm 1 sets *r* = *k* in equilibrium, and the fixed fee is positive.

6 It is important to remember that—in the Cournot context—even if the downstream rival has higher unit costs than the patentee, the rival will still produce (and sell) positive levels of this output. In this context, the patentee is better off licensing its cost-reducing innovation to the rival and thereby gaining some of the revenue that would otherwise accrue solely to the rival.

7 Note that, in the case of non-drastic innovation (*c* < 1/2), this happens even if the constraint is binding. Indeed, there is not excess capacity when *k* < (1 + c)/3, whereas the fixed fee is zero and the per-unit royalty is equal to *c* when *k* > *c*.

8 This extension refers to situations such as the recent “research collaboration and license agreement” by and between Pfizer Inc., Bionthec RNA Pharmaceuticals GmbH, and Bionthec AG (July 20, 2018): All firms produce, but Pfizer also rents its “unlimited” capacity to Bionthec RNA and Bionthec AG, which are capacity constrained.

9 Considering unit royalty excess capacity licensing, by altering the marginal cost of firm 1, makes the analysis unfeasible.

10 This happens both when firm 1 licenses its innovation and when it does not: In the former case, firm 1 benefits from the payments of the licensee; in the latter case, firm 1 benefits from its own lower marginal costs.

11 We do not explicitly consider differentiated Bertrand competition. However it should be noted that the results under a differentiated Bertrand duopoly where firms have different productions costs are often similar to asymmetric Cournot duopolies. Indeed, due to the existence of brand preferences in the case of product differentiation, the less efficient firm might survive in the case of price competition as under quantity competition. This is not possible when goods are homogenous, as the less efficient firm is driven out from the market in the case of price competition whereas it might survive in the case of quantity competition. We thank the Editor for raising this point.

12 See for instance Colombo and Filippini (2015).

13 Other types of licensing mechanisms that have received some attention in the recent patent licensing literature are ad valorem (or profit-sharing) royalty and revenue royalty; see Colombo and Filippini (2016); and Colombo et al., 2021). The analysis of the implications of capacity constraint on these licensing mechanisms is left for future research.

14 In this paper, we have taken the capacity to be exogenously given. It is easy to show that, if unlimited amounts of capacity can be acquired at no cost, the profits of firm 1 are strictly increasing with *k*: Firm 1 will acquire sufficient capacity so that the capacity constraint is not binding. Note that it is well-know that the innovator profits are larger in equilibrium when there is no capacity constraint: when the innovator is an insider. When *k* > 1 (Wang, 1998, model) the profits are (1 + 5*c – c*^2^)/9 with non-drastic innovation, and 1/4 with drastic innovation; when *k* = 0 (Kamien & Tauman, 1986), the profits are (2 – *c*)*c*/4. Of course, introducing some costs for acquiring capacity might change this result. The exploration for the implications of capacity-acquisition costs is left for future research.
